# Commentary: Associations between attention-deficit/hyperactivity disorder and allergic diseases: a two-sample Mendelian randomization study

**DOI:** 10.3389/fpsyt.2026.1847288

**Published:** 2026-07-06

**Authors:** Wenpei Qi, Chenjie Zhang, Shuqi Zhang, Liwei Liu, Yuyan Chen

**Affiliations:** Department of Pediatrics, The First Affiliated Hospital of Zhejiang Chinese Medical University (Zhejiang Provincial Hospital of Chinese Medicine), Hangzhou, China

**Keywords:** allergic diseases, attention-deficit/hyperactivity disorder, causal inference, genetic epidemiology, Mendelian randomization

## Introduction

The relationship between attention-deficit/hyperactivity disorder (ADHD) and allergic diseases has attracted increasing attention in clinical research (1, 2). We read with great interest the study by Zhang et al. (3), which applied a Mendelian randomization (MR) approach to investigate this association. The authors reported that genetically predicted ADHD was associated with a slight increase in the risk of allergic asthma in the inverse-variance weighted (IVW) analysis. However, results from other MR methods were less consistent. The study did not support the conclusion that ADHD could increase the incidence of allergic diseases. By applying an MR design, the study aimed to minimize residual confounding and reverse causation that often affect observational analyses (4). While the study provides valuable insights, several aspects of the analytical strategy and interpretation of the results merit further discussion.

## Interpretation of the MR results

We would like to comment on the interpretation of the results related to asthma. In the primary analysis, the IVW indicated a statistically significant association between ADHD and allergic asthma (OR = 1.0612, P < 0.05), whereas MR-Egger and weighted median analyses did not reach statistical significance. The authors therefore interpreted the evidence as not supporting a causal association. However, discrepancies across MR estimators are not uncommon, as these methods rely on different statistical assumptions and vary in their sensitivity to pleiotropy and invalid instruments (5). IVW typically provides the most precise estimate when instruments are valid, whereas MR-Egger and weighted median approaches often have lower statistical power. Thus, inconsistency across estimators may reflect methodological differences rather than necessarily indicating the absence of a potential causal relationship.

## Heterogeneity assessment

Clarification regarding heterogeneity would improve interpretation. In the ADHD– asthma analysis, the Cochran’s Q test for the IVW model produced a P value below 0.05, suggesting heterogeneity among the instruments. This was interpreted as indicating no heterogeneity, which may affect the reliability of the conclusions. In addition, the values reported for the Cochran’s Q test in Table 4 appear unusual, with a P value exceeding the valid range (0–1) (6), suggesting that the Q statistic and its corresponding P value may have been interchanged. Clarification of this point would help readers better interpret the heterogeneity assessment.

## Instrument selection and statistical power

To address potential bias, the authors performed sensitivity analyses using a more stringent SNP selection threshold (p < 5 × 10^-8^), which substantially reduced the number of instrumental variables. Although stricter thresholds may improve instrument validity, they can also reduce statistical power. In MR studies, instrument strength is typically assessed using the F statistic, and variants with F values greater than 10 are generally considered sufficiently strong to minimize weak-instrument bias (7). However, the original study reported a high overall F-statistic (F = 3729.4), suggesting that the initial instrument set was sufficiently strong. Therefore, further tightening the selection threshold may unnecessarily reduce the number of instruments and consequently the power of the analysis (8). Under such circumstances, a loss of statistical significance may reflect limited power rather than definitive evidence against a causal relationship.

## Conclusion

Taken together, the results suggest a modest association between genetically predicted ADHD and allergic asthma in the IVW analysis, although evidence from sensitivity analyses remains limited. Compared with conventional observational studies, the MR framework applied in this study is less susceptible to confounding and reverse causality, while also offering exploratory insights into the potential genetic and biological mechanisms underlying the observed association (9). A more cautious interpretation of the findings may help avoid potential over-interpretation while still acknowledging the strengths of the MR framework used in the study. We appreciate the authors’ contribution to this important area and hope these comments can help clarify the interpretation of MR findings in this field.
